# Malignant phyllodes tumor in Lynch syndrome: a case report

**DOI:** 10.1186/s13256-019-2138-0

**Published:** 2019-07-16

**Authors:** Shayma Kazmi, Steven Wagner, Rebecca Heintzelman, Melanie Corbman

**Affiliations:** Cancer Treatment Centers of America, Comprehensive Care and Research Center, 1331 East Wyoming Avenue, Philadelphia, PA 19124 USA

## Abstract

**Background:**

Lynch syndrome, or hereditary nonpolyposis colorectal cancer, is an autosomal dominant genetic syndrome that predisposes individuals to multiple cancer types. The known cancers associated with Lynch syndrome include colorectal and endometrial cancers as well as cancers of the stomach, ovary, urinary tract, hepatobiliary tract, pancreas, small bowel, and brain. There are no searchable cases of malignant phyllodes of the breast associated with Lynch syndrome.

**Case presentation:**

Our patient was a 43-year-old Caucasian woman who felt a lump in her left breast and was found to have a spindle cell neoplasm. Definitive surgery revealed a malignant phyllodes tumor. On the basis of her cancer diagnosis and family history of multiple cancers, a Myriad myRisk Hereditary Cancer® test panel of 25 genes was performed. This testing revealed that she had a heterozygous *MSH6* mutation as part of the Lynch syndrome panel. Due to positive margins, the patient received adjuvant chemotherapy with doxorubicin and ifosfamide. She also had a subsequent total abdominal hysterectomy and a bilateral salpingo-oophorectomy for risk reduction. She remains in a high-risk surveillance program. Her family members have been tested, which revealed that her two brothers and daughter also carry the genetic mutation.

**Conclusions:**

This case highlights the importance of genetic testing with rare malignancies because the full scope of phenotypic sequelae for known hereditary syndromes has not been mapped.

## Background

Lynch syndrome, or hereditary nonpolyposis colorectal cancer (HNPCC), is an autosomal dominant genetic syndrome that predisposes individuals to multiple cancer types and is caused by germline mutations in one of the mismatch repair genes, usually *MLH1* (human MutL homolog 1), *MSH2* (MutS protein homolog 2), *MSH6* (MutS protein homolog 6), or *PMS2* (PMS protein homolog 2) [1]. Affected individuals are highly susceptible to colorectal and endometrial cancers, as well as to cancers of the stomach, ovary, urinary tract, hepatobiliary tract, pancreas, small bowel, and brain. There are some *MSH6* and *PMS2* germline pathogenic variants implicated in breast cancer [2]. However, there are no reported cases of malignant phyllodes tumor of the breast with Lynch syndrome. The present case report describes an unusual presentation of Lynch syndrome and highlights the fact that there is much more to be learned about this genetic disease.

## Case presentation

**Fig. 1:**
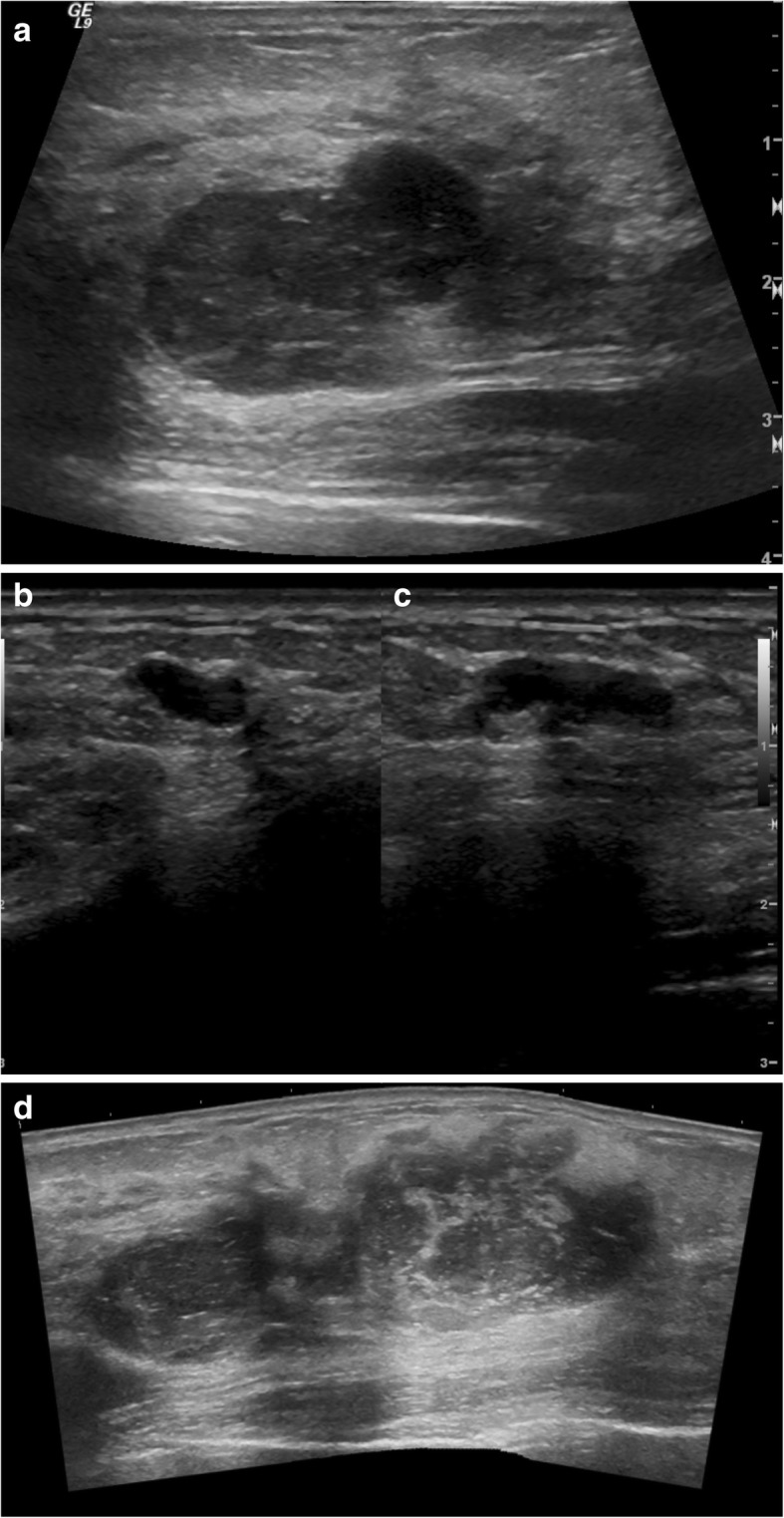
**a** Ultrasound image of left breast antiradial plane at 1:00 position 9 cm from the nipple demonstrates a mixed cystic and solid, irregularly shaped, hypoechoic, and heterogeneous mass with ill-defined margins and areas of posterior acoustic enhancement. Overall lesion size is 3.7 × 2.2 × 4.0 cm. **b** Ultrasound image of left breast antiradial plane at 12:00 position 8 cm from the nipple demonstrates a predominately solid, lobulated, hypoechoic, and heterogeneous mass with smooth margins and only minimal posterior acoustic enhancement. Overall lesion size is 3.4 × 1.8 × 2.7 cm. **c** Ultrasound image of left breast split screen antiradial (left) and radial (right) planes in 2:00 position 6 cm from the nipple demonstrates a predominately solid, lobulated, homogeneous, and hypoechoic mass with smooth, well-defined margins and areas of posterior acoustic enhancement. Overall lesion size is 0.8 × 0.4 × 1.2 cm. **d** Ultrasound image of the left breast with panoramic view shows the lesions shown in **a** and **b** may coalesce into a single larger lesion. This coalescent lesion is congruent with the pathologic findings of an 8.8-cm lesion

**Fig. 2:**
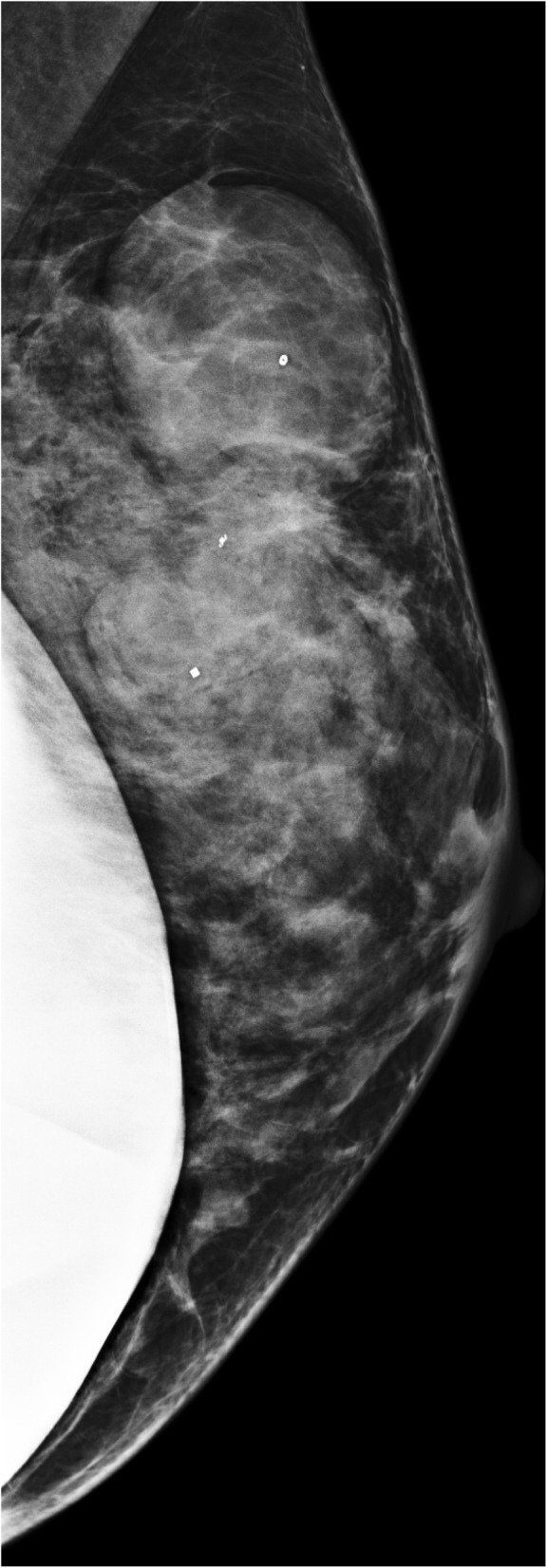
Post-biopsy mammography of the left breast left mediolateral projection performed in 2015 demonstrates rapid growth of lobulated, smoothly marginated, noncalcified masses

Our patient was a 43-year-old Caucasian woman reported a lump in her left breast and presented to her gynecologist in 2015. Notably, she had routine screening mammograms in 2014 and earlier in 2015 that were notable for dense breast tissue but otherwise unremarkable (Fig. 1a). Her physical examination revealed this mass to be “cystic” in nature but of fair size, measuring approximately 3 cm. She thereafter underwent a left breast ultrasound (Fig. 1) that showed three masses within the upper outer quadrant of the left breast. The 1:00 mass measured 3.7 × 2.2 × 4.0 cm; the 12:00 mass measured 3.4 × 1.8 × 2.7 cm; and the 2:00 mass measured 0.8 × 0.4 × 1.2 cm. Ultrasound-guided biopsy of all three lesions was performed. Three postbiopsy clips were present within the lesions (Fig. 2b).

Pathology revealed the following: (1) 1:00 spindle cell neoplasm, p63 (transformation-related protein 63), cytokeratin 5/6, and pan-keratin negative; (2) 12:00 fibroadenoma with prominent epithelial elements in uniform bland myxoid stroma; and (3) 2:00 fibroadenoma. Biopsies of the spindle cell neoplasm did not display significant cytologic atypia or mitotic activity. Differential diagnoses included mucoid hamartoma, myofibroblastoma, solitary fibrous tumor, and spindle cell lipoma. Malignant phyllodes tumor was less likely, owing to the low-grade nature of the patient’s lesions.

**Fig. 3:**
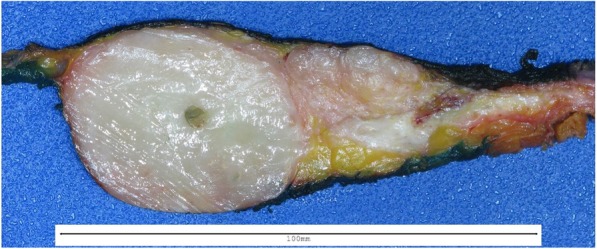
Left mastectomy with an 8.8 × 8.7 × 4.1-cm white-gray, rubbery mass spanning the 12 o’clock to 2 o’clock positions

**Fig. 4:**
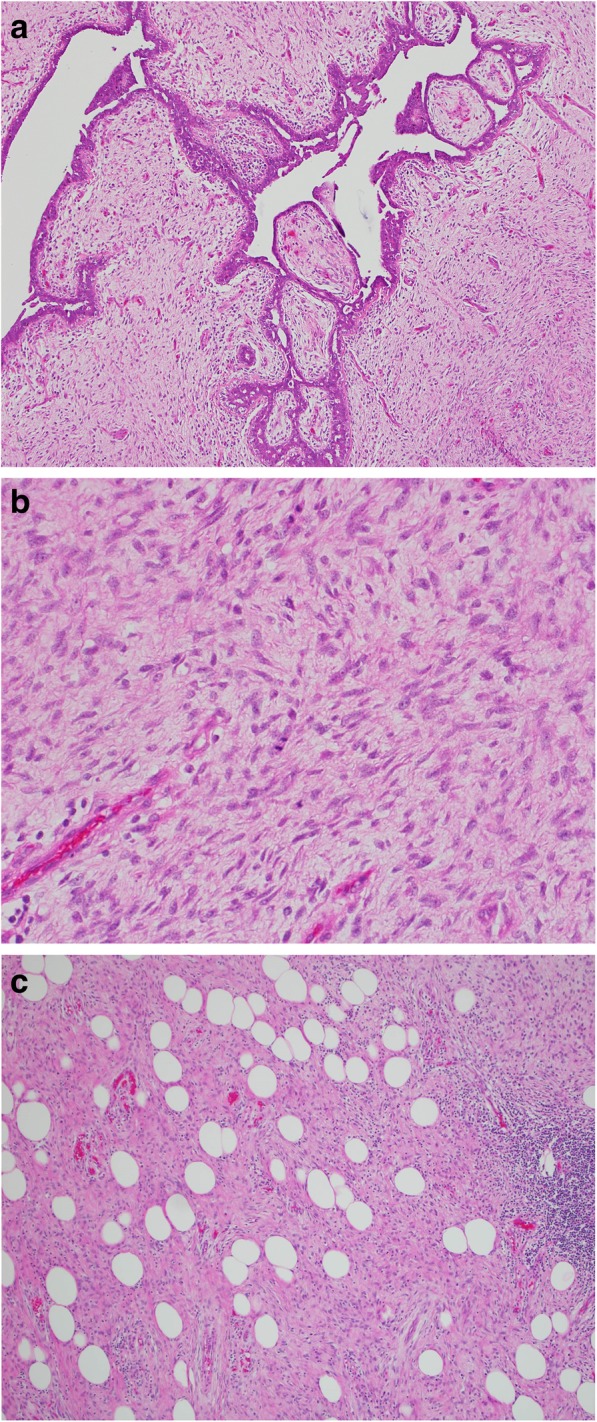
**a** Area of tumor with compression of the epithelium forming cleftlike spaces with a “leaflike” pattern. **b** Area of phyllodes tumor with stromal/sarcomatous overgrowth demonstrating nuclear atypia and increased mitotic activity. **c** Malignant phyllodes tumor with infiltrative growth pattern into the surrounding adipose tissue

**Fig. 5:**
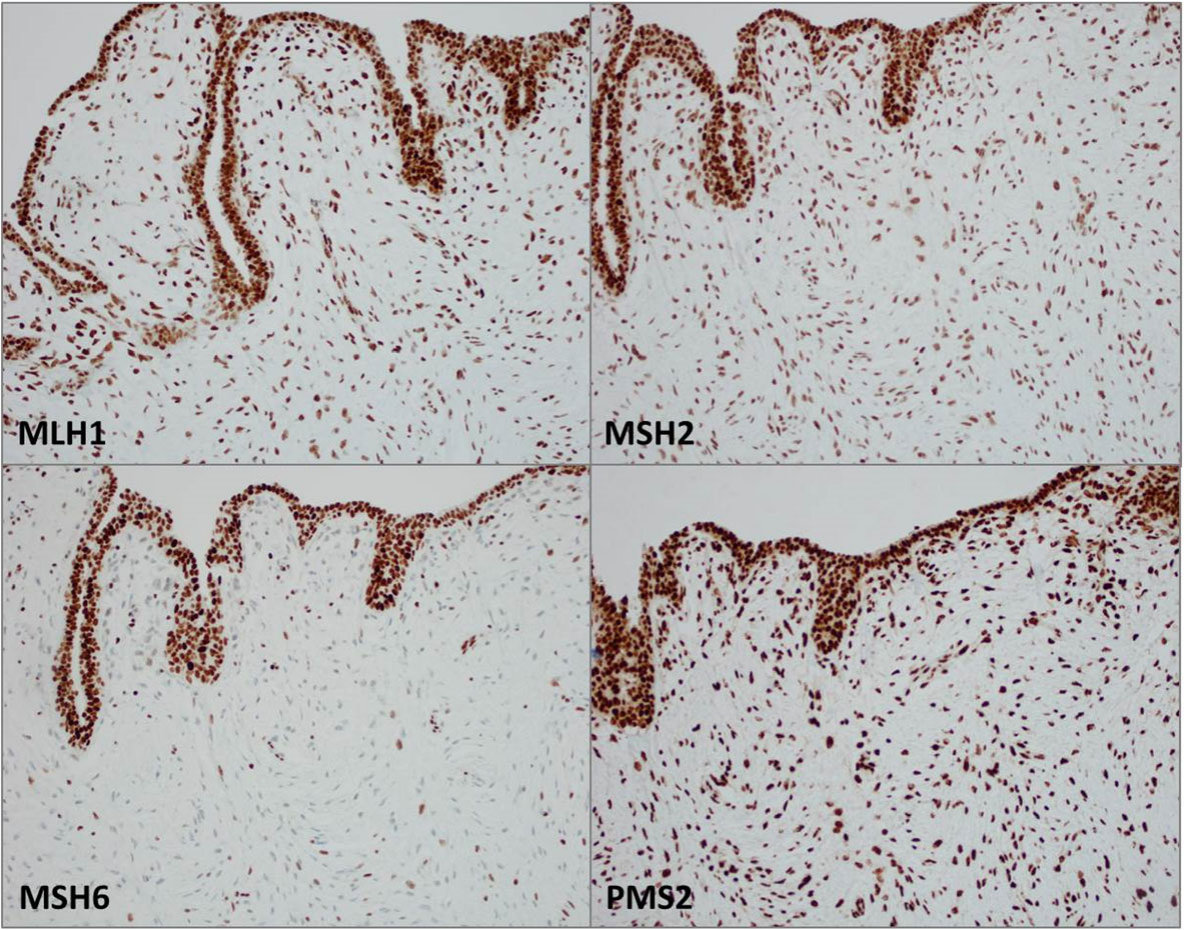
Mismatch repair protein evaluation showing intact nuclear expression within the epithelium and sarcomatous stroma for MLH1, MSH2, and *PMS2* proteins. *MSH6* protein showed intact expression in the epithelium with decreased expression in the sarcomatous stroma. Molecular testing for microsatellite instability was stable

After a multidisciplinary evaluation, the patient underwent left breast nipple-sparing mastectomy due to multiple large lesions in that breast with immediate expander placement and eventual implant. Pathology demonstrated an 8.8-cm, white-gray, rubbery mass in the upper outer quadrant (Fig. 3) consistent with a malignant phyllodes tumor showing some areas of leaflike proliferation (Fig. 4a) but with infiltrative borders (Fig. 4b) and sarcomatous stromal overgrowth with increased mitotic activity (Fig. 4c). The superior surgical margin was positive. Mismatch repair protein immunohistochemical staining showed intact protein expression for MLH1, MSH2, and *PMS2* with partial loss of protein expression for *MSH6* (Fig. 5). Molecular testing for microsatellite instability (MSI) was performed and was found to be stable.

The patient then underwent left chest wall radiation therapy from late 2015 in 25 fractions, which was complicated by an admission for cellulitis. Due to positive margins and extensive disease, adjuvant chemotherapy was administered. She had three cycles of doxorubicin and ifosfamide for 2 months in early 2016, complicated by chemotherapy-induced nausea/vomiting, thrombocytopenia, and leukopenia.

**Fig. 6:**
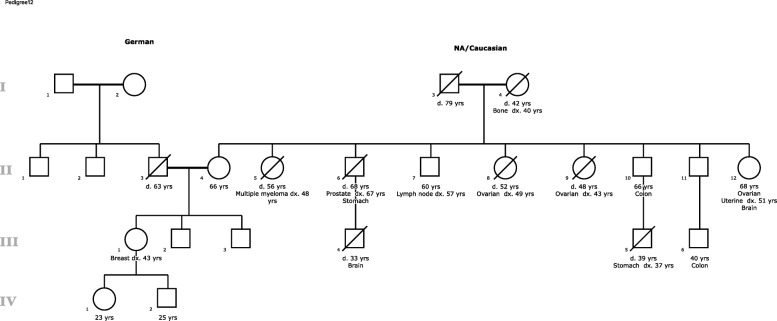
Family Pedigree

Genetic counseling was initially completed to help inform surgical decisions for her newly diagnosed breast cancer. On the basis of the size of her breast tumor, unilateral mastectomy was suggested. The patient wanted information on the risk for a contralateral breast cancer in order to decide whether she should have prophylactic mastectomy of the contralateral breast. At the initial counseling session, the patient reported a very strong maternal family history of colon, uterine, ovarian, stomach, and brain cancers suggestive of Lynch syndrome (Fig. 6). The patient underwent Myriad myRisk Hereditary Cancer® genetic testing in 2015. This panel was chosen on the basis of her young age of diagnosis of breast cancer and multiple family members with cancer diagnoses.

Although the patient was not found to carry a mutation in any of the genes associated with an increased risk for breast cancer, she was found to have an *MSH6* pathogenic mutation associated with Lynch syndrome/HNPCC. She underwent extensive counseling regarding her diagnosis. Her family was offered cascade testing for the identified mutation. As suggested by the family history, her mother had a positive test result for the *MSH6* mutation. Although her mother carries the *MSH6* mutation, she was cancer-free at age 65 after having had prophylactic total abdominal hysterectomy/bilateral salpingo-oophorectomy (TAH/BSO) at age 52, and had been undergoing screening with colonoscopy and endoscopic gastroduodenoscopy (EGD) based on her family history. The patient’s two brothers and daughter were also found to carry the identified *MSH6* mutation and were recommended to follow National Comprehensive Cancer Network guidelines for Lynch syndrome screening.

The patient underwent a prophylactic TAH/BSO a few months after completion of therapy in 2016, and the pathology was found to be benign. She is currently followed in a high-risk program and undergoes colonoscopy/EGD, capsule endoscopy, urine cytology, and skin examinations for screening.

## Discussion

Phyllodes tumors account for less than 1% of all breast neoplasms [3, 4]. The vast majority of phyllodes tumors occur in women, with a median age of presentation of 42 to 45 years [5–7]. Phyllodes tumors have been associated with Li-Fraumeni syndrome, a rare autosomal dominant condition that is characterized by the development of multiple tumors [8]. No other etiologic or predisposing factors have been linked to phyllodes tumors.

This case highlights an association between the diagnosis of malignant phyllodes tumor in a young woman who was ultimately diagnosed with Lynch syndrome. Lynch syndrome is an autosomal dominant genetic disorder and significantly increases risk of cancers of the colon/rectum, uterus, ovary, stomach, small bowel, hepatobiliary system, renal pelvis, ureter, and brain, as well as the risk of sebaceous neoplasms. Rare associations with breast cancers have been reported recently. No reported cases of sarcomas or malignant phyllodes tumors were discovered in a PubMed literature search. Current screening guidelines for patients with Lynch syndrome include heightened surveillance for gastrointestinal malignancies with more frequent upper and lower endoscopies [9, 10]. Women should also undergo screening for endometrial and ovarian cancers, with optional prophylactic hysterectomy/oophorectomy after childbearing has completed or at age 40. Annual urinalysis for cytology as well as dermatological and neurological examinations are also recommended [9]. Currently, annual mammograms are advised for women over the age of 40, regardless of their genetic predisposition.

Phyllodes tumors are subcategorized histologically by the World Health Organization as benign, borderline, or malignant, according to the degree of stromal overgrowth, cellular atypia, and mitotic rate. Approximately 20% of phyllodes tumors present as a nonpalpable mass identified by screening mammography [11]. There is substantial overlap of imaging findings between benign, borderline, and malignant phyllodes tumors. Mammography may demonstrate a dense, nonspiculated, and lobulated mass. Figure 2 demonstrates development of our patient’s left breast masses over the course of 15 months as visualized by mammography. This rapid growth of breast masses has been suggested to be a common finding in malignant phyllodes tumors [12]. Ultrasound features are variable and can include irregular or oval/rounded mass, with circumscribed or noncircumscribed margins, heterogeneous or homogeneous hypoechoic pattern, and with or without posterior acoustic enhancement. Given the overlap of imaging findings among phyllodes tumors, the majority of mammographic and ultrasound features have been unable to differentiate between benign and malignant phyllodes tumors [13, 14]. The only imaging feature shown to be associated with a statistically increased likelihood of malignancy is size 3 cm or greater [13]. Studies evaluating breast magnetic resonance imaging (MRI) have also determined that MRI cannot reliably differentiate between a fibroadenoma and a phyllodes tumor [15].

As in our patient’s case, ultrasound demonstrated three lesions in which the largest lesion measured up to 4.0 cm. Potentially, two of the three lesions may have coalesced into the larger lesion identified on pathologic assessment of the surgical specimen. The lack of MSI within the tumor despite a known germline mutation in *MSH6* and heterogeneous expression by immunohistochemistry is not an uncommon finding. Although the *MSH6* gene is a component of the DNA mismatch repair, some tumors with germline mutations in *MSH6* may not show MSI-high disease. Instead, they tend to show a lower level of MSI: either MSI-low or microsatellite-stable tumors [16, 17]. A proposed explanation for this is that the major function of *MSH6* is thought to be in the correction of base-base mismatches, which do not give rise to MSI [18]. The lack of MSI has also been thought to be a consequence of the partial redundancy of the function of *MSH6* and MSH3 proteins [17, 19].

Although no specific changes in current screening or monitoring guidelines for patients with Lynch syndrome need to be made, it is evident that these hereditary syndromes may harbor propensity for more cancers than previously noted. We recommend genetic counseling for any patient who harbors a rare cancer, because the link to genetic syndromes is not yet definitive owing to the paucity of cases.

## Data Availability

The datasets used and/or analyzed during the current study are available from the corresponding author on reasonable request.

## Abbreviations

EGD: Endoscopic gastroduodenoscopy
HNPCC: Hereditary nonpolyposis colorectal cancer
MLH1: Human MutL homolog 1
MRI: Magnetic resonance imaging
MSH2: MutS protein homolog 2
MSH6: MutS protein homolog 6
MSI: Microsatellite instability
p63: Transformation-related protein 63
PMS2: PMS protein homolog 2
TAH/BSO: Total abdominal hysterectomy/bilateral salpingo-oophorectomy
